# Supplementary material for: Complex‐centric proteome profiling by SEC‐SWATH‐MS
Source: Mol Syst Biol. 2019 Jan 14;15(1):e8438. doi: 10.15252/msb.20188438 (PMC6346213; doi:10.15252/msb.20188438)
Supplement: Supplementary file 8 — Dataset EV7 [file MSB-15-e8438-s008.zip › feature_plots_string/P78316.pdf]

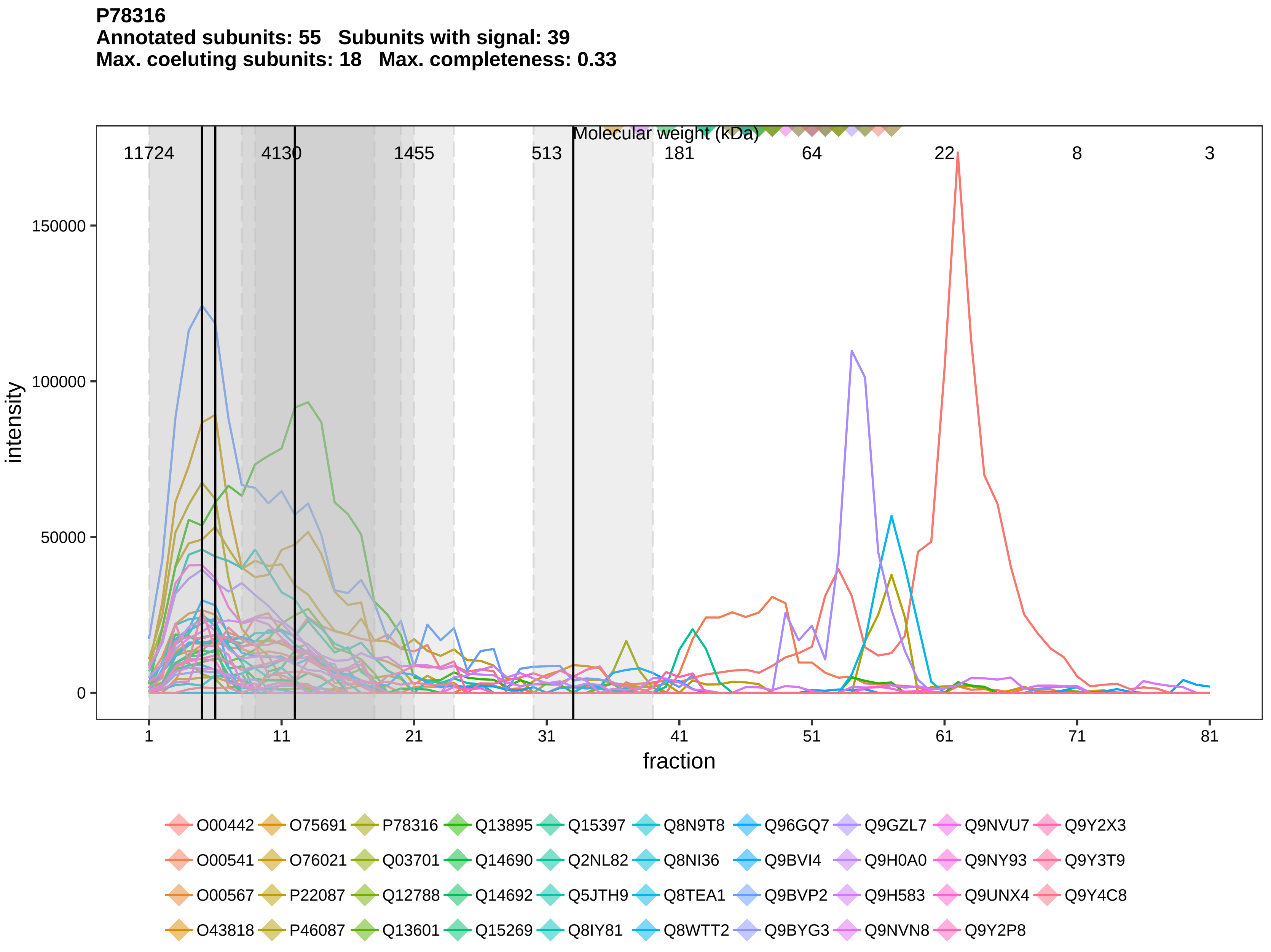

P78316
Annotated subunits: 55 Subunits with signal: 39
Max. coeluting subunits: 18 Max. completeness: 0.33
Molecular weight (kDa)
11724
4130
1455
513
181
64
22
8
3
intensity
150000
100000
50000
0
1
11
21
31
41
51
61
71
81
fraction
O00442
O00541
O00567
O43818
O75691
O76021
P22087
P46087
P78316
Q03701
Q12788
Q13601
Q13895
Q14690
Q14692
Q15269
Q15397
Q2NL82
Q5JTH9
Q8IY81
Q8N9T8
Q8NI36
Q8TEA1
Q8WTT2
Q96GQ7
Q9BVI4
Q9BVP2
Q9BYG3
Q9GZL7
Q9H0A0
Q9H583
Q9NVN8
Q9NVU7
Q9NY93
Q9UNX4
Q9Y2P8
Q9Y2X3
Q9Y3T9
Q9Y4C8
